# Role of behavioural interventions in managing gestational weight gain in overweight and obese pregnant women: A Scoping Review

**DOI:** 10.1007/s00404-025-08144-x

**Published:** 2025-09-03

**Authors:** Rukmini Padiyar, Jyothi Shetty, Sanya Anklesaria, T. S. Shwetha, Shripad Hebbar, G. Shyamala, Suvarna Hebbar, Preetha Ramachandra

**Affiliations:** 1 Department of Physiotherapy, Manipal College of Health Professions, Manipal Academy of Higher Education, Manipal, Karnataka India; 2 Department of Obstetrics and Gynaecology, Kasturba Medical College, Manipal, Manipal Academy of Higher Education, Manipal, Karnataka India; 3 Department of Clinical Psychology, Manipal College of Health Professions, Manipal Academy of Higher Education, Manipal, Karnataka India; 4 Department of Clinical Nutrition and Dietetics, Manipal College of Health Professions, Manipal Academy of Higher Education, Manipal, Karnataka India

**Keywords:** Behavioural interventions, Gestational weight gain, Obese, Overweight, Pregnancy

## Abstract

**Purpose:**

Women undergo various physiological, psychological, and hormonal changes during pregnancy. Approximately, two-thirds of expecting mothers gain excessive gestational weight beyond the recommended guidelines. Dietary habits, physical activity, and lifestyle choices can contribute to excessive gestational weight gain. Behavioural interventions encourage a healthy lifestyle by focusing on changing people’s actions to improve their health. Pregnant women who are overweight or obese may benefit from behavioural intervention in addition to dietary and exercise changes. The purpose of the review is to determine the impact of behavioural intervention on gestational weight gain in overweight and obese pregnant women.

**Methods:**

A scoping review was conducted using Medline, Web of Science, Scopus, Embase, and ProQuest. Two independent review authors screened the title, abstract, and full text which met the inclusion criteria. The relevant studies and their reported outcomes were organized and analysed thematically.

**Results:**

The search yielded 4704 records; 4089 entries were found after removing duplication and screening for title and abstract. This scoping review includes 8 of the 69 papers that were chosen for full text. The behavioural intervention was delivered through face-to-face in-depth counselling, followed by telephonic counselling about physical activity, dietary intake, and appropriate weight gain during pregnancy.

**Conclusions:**

The review concluded that to achieve appropriate GWG, a unique approach that promotes a healthy diet and exercise through behavioural intervention is required. Adopting innovative strategies along with increasing the duration of interaction with pregnant women may result in behaviour change, which may facilitate adequate gestational weight gain.

## Introduction

Pregnancy is a phase in a woman’s life that is characterized by physiological and hormonal changes that occur to support and protect the mother and growing foetus and help the mother prepare for delivery [1]. One of the physiological changes is the gestational weight gain that occurs during pregnancy. Increasing body weight is an indication of the growth of the foetus and maternal adaptation [2]. Gestational weight gain (GWG) is the total weight gained during pregnancy and is measured as the difference between the last measured body weight before childbirth and the weight measured at the time of conception [3].

Overweight and obesity have been increasing in pregnant women in the past few decades and have become a major health concern [4, 5]. According to Institute of Medicine (IOM) guidelines, pregnant women who are overweight (body mass index {BMI} ≥ 25–29.9 kg/m^2^) can gain up to 7–11 kg, and pregnant women who are obese (BMI > 30 kg/m^2^) can gain up to 5–9 kg, as this reduces the risk of adverse pregnancy outcomes in both the mother and the baby. In Asia, about 10% of pregnant women are overweight and obese, and approximately 37% gain excessive gestational weight [5].

Excessive GWG can lead to adverse maternal outcomes such as gestational diabetes, gestational hypertension, increased chances of caesarean delivery, lower rate of initiation of breastfeeding, and postpartum weight retention. In the long term, excessive GWG can lead to metabolic consequences such as type 2 diabetes, metabolic syndrome, and cardiovascular disease. In neonates, excessive GWG in the pregnant mother can lead to macrosomia, childhood obesity, and chronic diseases such as hypertension, diabetes, and other metabolic disease in the later stage of life [6].

The factors for excessive GWG are many, but the major modifiable factors are dietary intake and physical activity during pregnancy. Increased calorie intake, along with a higher proportion of protein and lipids of animal origin and intake of a lower proportion of carbohydrate, is associated with increased GWG in the second trimester [7]. Increasing physical activity during pregnancy has a stronger influence on optimal GWG, as physical inactivity is a major contributor to excessive GWG [8]. Physical activity and strengthening exercises during pregnancy are shown to have a beneficial effect on maternal and foetal health [9].

Supervised antenatal exercise prevents excessive gestational weight gain in overweight and obese pregnant women, but there is a lack of adherence to exercises among pregnant women [10, 11]. A previous study has reported that the lack of adherence to exercise could be due to the perception that exercises may harm the foetus, feeling uncomfortable during training, and lack of support from the partner and family [12]. Previous literature indicates that a novel strategy should be considered in overweight and obese pregnant women to improve adherence to the prescribed exercise programme and a novel strategy that is deeply rooted in behavioural interventions was recommended [13].

Behavioural interventions change a person’s maladaptive behaviour and focus on changing the actions people take to improve their health [13]. The intervention includes psychoeducation, cognitive behaviour therapy [14], motivational interviews [15], counselling [16], and lifestyle management [17]. Through behavioural interventions, women can be encouraged to maintain a healthy lifestyle throughout their pregnancy [13]. Thus, the review aims to find the gaps in the literature and determine how behavioural interventions influence gestational weight gain in overweight and obese pregnant women.

### Objective of the review

The review facilitates determining the impact of behavioural intervention on gestational weight gain in overweight and obese pregnant women.

### Research question

Objectively, the research focused on understanding the role of behavioural intervention on gestational weight gain among overweight and obese pregnant women.

## Methodology

The review was carried out under the guidelines set forth by the Joanna Briggs Institute (JBI), which are based on Arksey and O’Malley and Levac et al. [18, 19], for scoping reviews. It has five steps, as follows:

## (1) Search strategy: identifying relevant studies

A comprehensive and systematic search was carried out from inception to March 2025, with the following databases: MEDLINE, WEB OF SCIENCE, SCOPUS, EMBASE, and PROQUEST; only published English articles were eligible for the review. The details of the inclusion and exclusion criteria are given in Table 1.

The following keywords were used for the search strategy:

((((Gestational weight gain OR Gestational Pregnancy Weight Gain OR Weight Gain Pregnancy OR Maternal Weight Gain OR Weight Gain OR Maternal Antepartum Weight Gain OR Weight Retention OR Antenatal weight gain) AND (Overweight OR Obese OR Obesity OR High Body Mass Index)) AND (Pregnant Woman OR Woman OR Pregnant Women OR Pregnant OR Pregnancy OR Obstetric)) AND (Antenatal OR Prenatal OR Antepartum)) AND (Behavioural intervention OR Behaviour Therapies OR Behaviour Treatment OR Behaviour Therapy OR Conditioning Therapy OR Conditioning Therapies OR Behaviour Change Techniques OR Behaviour Change Technique OR Behaviour Modification OR Behaviour Modifications OR Life style management OR alter behaviour OR behaviour analysis OR Cognitive Behaviour Therapy OR Cognitive behavioural counselling OR Psychological interventions OR Self efficacy OR Self-regulation Or behaviour maintenance therapies Or behaviour change approaches OR Self-monitoring OR goal setting OR problem-solving OR relapse prevention OR reinforcement OR Modelling Or Motivational interviewing OR wellbeing OR quality of life OR relaxation).

## (2) Study selection

The Rayyan software was used to screen and select the studies using the above-mentioned keywords and database. For the multiple reports of the same primary study or duplicate publications, we used the most comprehensive dataset that is compiled from all known publications to maximize the information yield (Table 1).

**Table 1:** Inclusion and exclusion criteria in the PICOT framework

Elements of the PICOT framework	Inclusion criteria	Exclusion criteria
Type of study	Randomized/nonrandomized/cluster trials were included	Only abstract Conference papers
Participants	Pregnant women over the age of 18 years Pregnant women who were overweight or obese at the time of conception	Studies involving animals
Interventions	Studies delivering behavioural interventions with a focus on diet and exercise	Studies involving interventions for psychological disorders
Comparisons	Control/comparator/alternate treatments/usual care/education	
Outcome measures	Studies that use gestational weight gain as one of their outcome indicators	

## (3) Charting the data

The title and abstract screening were done following the inclusion and exclusion criteria and the full text was independently evaluated by two independent reviewers (RP and SA). When a consensus was not reached, a third researcher (PR) resolved the disagreement.

## (4) Collating and summarizing the result

The PRISMA flow diagram was used to report the findings of the search strategy and selection process. The studies that met the inclusion criteria were included in the standardized data extraction sheet. The data extraction sheet includes author, year, country, aim(s), study population, type of intervention, methodology, strength, limitation, and conclusion. Findings are reported as a narrative synthesis.

## (5) Consultation

The search team consists of multiple disciplines such as Women’s health physiotherapists, obstetrician and gynaecologist, and a clinical psychologist. For analysis of the literature, this diverse group contributed with a range of experiences and viewpoints.

## Results

**Fig. 1:**
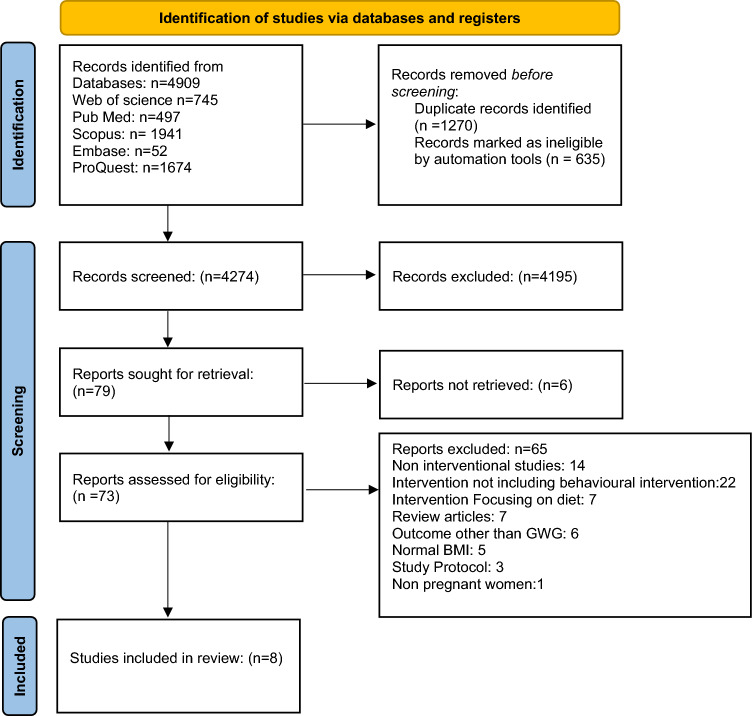
PRISMA flowchart

The search led to the identification of 4704 records, and after duplication, 4089 records were identified and screened for title and abstract. Of these, 69 full-text articles were selected and eight articles were included in the scoping review. The PRISMA flowchart is represented in (Fig. 1).

The selected articles in the scoping review are from the year 2002–2025. At the time of recruitment, the gestational age ranged between 12 and 16 weeks. In this review, it was found that the behavioural interventions commonly reported in the literature were face-to-face in-depth counselling [4, 20–24] and telephonic counselling [5, 25] about physical activity, dietary intake, and appropriate weight gain during pregnancy. All eight studies measured gestational weight gain as one of the outcome measures. Studies also investigated other outcome measures such as physical activity, dietary intake, and maternal and neonatal outcomes (Table 2) [4, 5, 20–22, 24–26].

**Table 2:** Study characteristics

Author/year	Objective	BMI included	Study design	Participants	Outcomes
Polley et al., 2002, [26]	Developing and investigating a stepped-care behavioural intervention in reducing excessive weight gain	Normal BMI = 19.8–26 kg/m^2^ Overweight BMI = 26.1–29 kg/m^2^	Randomized controlled trial	N = 110, Intervention group = 57 (30 normal weight, 27 overweight) Control group = 53 (31 normal weight, 22 overweight)	Excessive weight gain during pregnancy. Pregnancy complications. Foetal outcomes. Postpartum weight loss and weight retention
Phelan et al., 2011, [24]	Decreasing the gestational weight gains in the proportion of women who exceeded the 1990 Institute of Medicine (IOM) recommendations and increasing the proportion of women who returned to pregravid weight by 6 months postpartum through behavioural intervention	BMI 19.8–40 kg/m^2^	Randomized controlled trial	N = 401, intervention group = 200, control group = 201	Proportion of women with excessive gestational weight gain Pregnancy complications. Foetal outcome
Liu et al., 2015 [22],	Evaluating feasibility and satisfaction of a theory-based diet and physical activity intervention in preventing excessive GWG, encouraging weight loss in the postpartum period among African-American women who were overweight or obese	BMI between 25.0 and 40.0 kg/m^2^	A pilot theory-based randomized controlled trial	Pregnant (n = 25) and postpartum (n = 8)	Gestational weight gain Postpartum weight retention Physical activity (PA): SenseWear Armband Dietary intake: 24-h recall Programme satisfaction: structured, open-ended question
Chao et al. 2017 [25],	Comparing a lifestyle modification to treatment as usual (TAU) in overweight or obese women delivered via phone for the prevention of excess gestational weight gain	BMI between 25 and 50 kg/m^2^	Pilot randomized controlled trial	N = 41 Intervention = 20 Treatment as usual = 21	Gestational weight gain Maternal and neonatal birth outcome
Kunath et al., 2019, [21]	Effect of a lifestyle intervention on the proportion of pregnant women with excessive GWG and obstetric complications and long-term risk of maternal and infant obesity	BMI ≥ 18.5 kg/m^2^ and ≤ 40.0 kg/m^2^	Randomized controlled trial	N = 2286 Intervention group = 1152 Control group = 1134	Excessive GWG Incidence of GDM Obstetric outcome Neonatal outcome
Ferrara et al., 2020, [5]	Telehealth lifestyle intervention in reducing excess GWG among women with overweight or obesity	BMI between 25·0 kg/m^2^ and 40·0 kg/m^2^	Randomized controlled trial	N = 400 Intervention group = 200 Usual care = 200	GWG Total caloric intake Perinatal complication Physical activity: self-reported and accelerometer
Darvall et al., 2020, [20]	Role of behavioural intervention in reducing excessive GWG and feasibility of self-monitoring of activity levels via the Fitbit Zip pedometer	BMI ≥ 30 kg/m^2^	Randomized controlled feasibility trial	N = 30 Control n = 10 App group n = 10 App couch group n = 10	Activity data Step count: Fitbit Zip pedometer GWG
Liu et al. 2021 [4],	Effect of behavioural lifestyle intervention on total GWG in White and African-American women with overweight or obesity	BMI ≥ 25 kg/m^2^	Theory-based randomized controlled trial	N = 228 Intervention = 114 Standard care = 114	GWG Physical activity: SenseWear Armband Dietary intake: 24 h dietary recall Maternal and infant health outcomes

A previous study assessed the effect of stepped-care behavioural intervention on excessive gestational weight gain during pregnancy. The standard group received nutritional counselling from physicians, nurses, nutritionists, and women, infants, and children’s (WIC) counsellor. The counselling focused on eating a balanced diet and recommended taking multivitamins and iron supplements. The intervention included written or oral information on healthy eating, exercise, and appropriate weight gain. Biweekly newsletters on healthy eating and exercise habits were mailed to the participants. Pregnant women exceeding recommended weight gain were given an individual stepped-care approach. The stepped-care approach included individual counselling on the review of weight gain chart, assessment of current eating habits with periodic computerized nutrition analysis, review of progress towards behavioural goals, problem-solving, instruction on the use of behavioural techniques such as stimulus control or self-monitoring, and goal setting for eating and exercise behaviours. The result showed that there was a significant difference in weight gain in pregnant women with normal BMI following the intervention, but there was no effect on gestational weight gain in overweight pregnant women. Thus, the study suggested that stepped-care intervention is beneficial in pregnant women with normal BMI and not for overweight pregnant women [26].

A study was conducted to assess the effect of behavioural lifestyle intervention delivered during pregnancy to reduce gestational weight gain in the proportion of women who exceeded recommendations. The standard group received prenatal care, every month until 28 weeks, every 2 weeks from 28 to 36 weeks, every week until birth, and every 6 weeks after delivery. Prenatal care included standard nutrition counselling provided by physicians, nurses, nutritionists, and counsellors from the Women, Infants, and Children’s state programme. Pregnant women were weighed by nurses at each visit; furthermore, a 15-minutes in-person visit with an interventionist and study newsletter that consisted general information about pregnancy-related issues during pregnancy and the postpartum period were provided. The intervention included one face-to-face visit that discussed the importance of increasing physical activity that involved walking for 30 minutes most of the days in a week, reducing high-fat foods, and daily self-monitoring of diet, exercise, and weight. Weekly mail on eating healthy, and exercise habits was sent to pregnant women. Additionally, pregnant women received personalized graphs of their weight gain with feedback. All participants received three supportive phone calls from the dietitian for 10–15 minutes. Pregnant women who are overweight and underweight received two supportive phone calls at one-month intervals with structured meal plans and specific goals, until appropriate weight gain was achieved. The study showed that there was no significant reduction in excessive gestational weight gain in overweight and obese pregnant women. The study concluded that self-reported pre-pregnancy weight was the major problem throughout the study, which led to incorrect classification of BMI of pregnant women. This suggests that intervention targeting diet, physical activity, and behavioural strategies are required to achieve ideal gestational weight gain in overweight and obese pregnant women [24].

A study assessed the feasibility and participant satisfaction of a theory-based nutrition and physical activity intervention designed to prevent excessive GWG and promote weight loss in the early postpartum period in overweight and obese African-American women. The face-to-face intervention was based on social cognitive theory including three 24-h dietary recalls and measurement of physical activity levels using a SenseWear Dietary Armband. The intervention also included personalized feedback on gestational weight gain, 90-minutes group sessions which included skill training in diet and/or physical activity, application of a behavioural strategy, and a group activity. The intervention telephone call evaluated health and safety and progress towards the physical activity and dietary goals. The result showed that the intervention group were less likely to exceed gestational weight gain based on IOM guidelines, and gained less weight. Thus, the study suggested that more intense intervention with more frequent contact and awareness on importance on both physical activity and nutrition is recommended for overweight and obese pregnant women [22].

A study assessed the feasibility and preliminary effect of an intervention using telephone counselling, which focused on limiting excess weight gain during pregnancy. The treatment as usual (TAU) control group, received counselling on nutrition, exercise, and weight gain goals provided by their obstetrician. The intervention consisted of weekly telephonic sessions that included weight control advice through nutrition and exercises. The session also included modification strategies to improve compliance with diet and physical activity. Pregnant women were asked to weigh themselves through a Wi-Fi scale. The Wi-Fi scale is a weight monitoring scale used in the study that transmitted participant’s weights to personalized weight charts accessed through the Internet by study investigators to give feedback to participants. There was no significant difference in GWG between the groups, as pre-pregnancy weight was self-reported which led to difficulty in weight classification. The study concluded that behavioural nutrition counselling through phone calls along with Wi-Fi scale must be started early in pregnancy. However, the ideal treatment component, delivery of treatment, and intensity of treatment must be determined in future research [25].

A previous study assessed the effect of a lifestyle intervention during pregnancy on the proportion of women with excessive GWG. The control group received routine prenatal care and information leaflets on a healthy lifestyle during pregnancy. The intervention consisted of face-to-face counselling, encouraging pregnant women to engage in physical activity, consume a balanced diet, and to self-monitor their weight. Weight gain chart along with recommended weight gain based on their BMI according to IOM guidelines was provided to the participants. The result showed that there was no statistical difference between the groups. Overweight pregnant women gained excessive weight compared to obese pregnant women. In this study, it was reported that there was higher adherence in the intervention group and pregnant women demanded additional counselling sessions. The counsellor was trained only for 2 days, which was not sufficient for high-quality coaching. It was concluded that trained and specialized counselling by health professionals would improve the quality of counselling and improve excessive GWG [21].

A randomized controlled trial assessed the feasibility of self-monitoring of physical activity levels through the Fitbit Zip pedometer and behavioural intervention in reducing excessive GWG. Participants were given a Fitbit Zip pedometer which was worn daily on the waistband and was synced with their smartphones. In the control group, the pedometer display was masked so that the participants were unaware of their daily step count and the duration for which they remained active. The pedometer of the participant was not synced to their smartphone. There were two intervention groups in this study: a) App group, and b) App Couch group. App groups were advised to wear a pedometer that was synced to the smart phone of pregnant women. App couch group received 1 h of face-to-face counselling session between 16 and 20 weeks and specific, measurable, achievable, relevant, and time-bound (SMART) goals were set. This was followed by three telephonic sessions for 20 minutes at 24 weeks, 28 weeks, and 32 weeks of gestation. The aim was to educate pregnant women on the importance of physical activity, healthy eating during pregnancy, balance between calorie intake and expenditure, removing myths, and improving confidence about physical activity during pregnancy. The results showed that there was no significant difference between the groups in GWG, as the sample size was small. The study reported that a pedometer synced with a smartphone may be feasible in improving physical activity. This suggests that a focus on improving compliance with wearable devices and engagement of wearables with intervention is necessary [20].

Another study assessed the effect of telehealth lifestyle intervention in reducing excess GWG among overweight or obese pregnant women. The usual care received standard Kaiser Permanente Northern California (KPNC) antenatal medical care that included antenatal visits, at 7–10 week of gestation, a periodic health education newsletter that included IOM guidelines, information on physical activity and healthy eating in pregnancy, and an additional seven antenatal visits. In addition to these, four study newsletters that focused on women’s health and safety during pregnancy without addressing GWG were provided to the usual care. The intervention administered by the dietician was based on the transtheoretical model, motivational interviews, and stepwise behavioural change based on social cognitive theory. Participants received a scale to encourage self-weighing, a printed workbook to discuss at each session, and a customized electronic or paper-based graph to monitor their weight. The in-person counselling included healthy eating, physical activity, weight management, and stress management. The intervention included 13 weekly sessions in which the first and the last sessions were conducted in person and the remaining sessions were conducted through telephonic counselling. The result showed that women in the intervention group had lower rates of weekly GWG and total GWG. The group difference in GWG between obese and overweight women was greater in obese women, suggesting that intervention was more effective in obese than overweight women. The study was successful in delivering intervention due to frequent follow-ups, the use of telehealth, and the use of electronic health records [5].

A previous randomized control trial which was based on social cognitive theory included in-depth counselling about weight gain, physical activity, and diet intake. Control groups were encouraged to attend prenatal care. The group received six monthly mailings and ten weekly podcasts both focusing on healthy pregnancy and foetal development. The intervention was based on social cognitive theory that consisted of in-depth counselling, followed by ten weekly phone calls and ten podcasts received by participants. Participants in the counselling session were required to plot their weight on a graph and discuss one behavioural approach, nutrition, and physical activity. Following the counselling calls, participants received shorter weekly or biweekly counselling calls that included behavioural goal setting for the following week, assessing changes in health status, discussing progress towards physical activity and healthy eating goals set in the previous call, resolving obstacles to achieving goals when necessary, and plotting weight. The results showed that total GWG in the intervention group was more favourable than in standard groups. The treatment effect among overweight participants for total GWG was less compared to the standard group. The study also reported that pregnant women who received intervention via telephonic calls showed a significantly favourable weight gain. It was suggested that effective strategies must be incorporated for healthy GWG in obese pregnant women [4].

## Discussion

The results of the scoping review demonstrated that behavioural intervention focusing on diet and physical activity improves GWG in overweight and obese pregnant women when administered by face-to-face counselling with telephonic follow-ups. The review demonstrated that behavioural intervention combined with technologies such as wearables for activity trackers, step count, or weight monitoring apps shows a better outcome.

A study that combined intervention focusing on healthy eating and physical activity along with face-to-face counselling reported that there was a significant reduction in GWG among obese pregnant women [27]. A previous meta-analysis reported that intense in-person counselling focusing on diet and physical activity resulted in lowering the excess gestational weight gain [28]. A study on in-person counselling on exercises reported that counselling helps in understanding the benefits of exercises during pregnancy and reducing adverse physiological and psychological symptoms. It also has a positive influence on pregnancy and promotes positive exercise behaviour [29]. Another study on dietary counselling in reducing excessive gestational weight gain resulted higher proportion of women gaining adequate body weight within the recommended IOM guidelines. Dietary counselling benefits pregnant women by improving food intake and helping to change dietary habits during pregnancy. Education on dietary intake and healthy weight gain must be incorporated regularly during pregnancy as it reduces excessive gestational weight gain [30].

Behavioural intervention is effective with an increased number of follow-ups and a greater number of counselling sessions that may help pregnant women to gain appropriate gestational weight [31]. Interventions which involved regular telephonic follow-up calls resulted in improvement in GWG, as these provided reminders and education to pregnant women and helped in improving their physical activity. The use of telephones or technologies helped women to stay motivated and change their behaviours toward health. The adherence rate to the intervention was found to be reduced in the overweight and obese population. However, an interdisciplinary approach that includes behavioural interventions along with a personalized diet and a physical activity programme may benefit this population [32, 33].

## Conclusion

The scoping review assessed the impact of behavioural intervention on gestational weight gain among overweight and obese pregnant women. The review concluded that to achieve appropriate GWG, a unique method utilizing behavioural interventions which include face-to-face counselling or telephonic counselling that emphasizes the importance of diet and physical activity is needed. Extensive interaction with pregnant women through behavioural interventions may bring a change in their lifestyle and help in attaining the recommended gestational weight gain among overweight and obese pregnant women.

## Data Availability

No datasets were generated or analysed during the current study.
